# Supplementary figures and images for: Human Mesenchymal Stromal Cells Unveil an Unexpected Differentiation Potential toward the Dopaminergic Neuronal Lineage
Source: Int J Mol Sci. 2020 Sep 9;21(18):6589. doi: 10.3390/ijms21186589 (PMC7555006; doi:10.3390/ijms21186589)

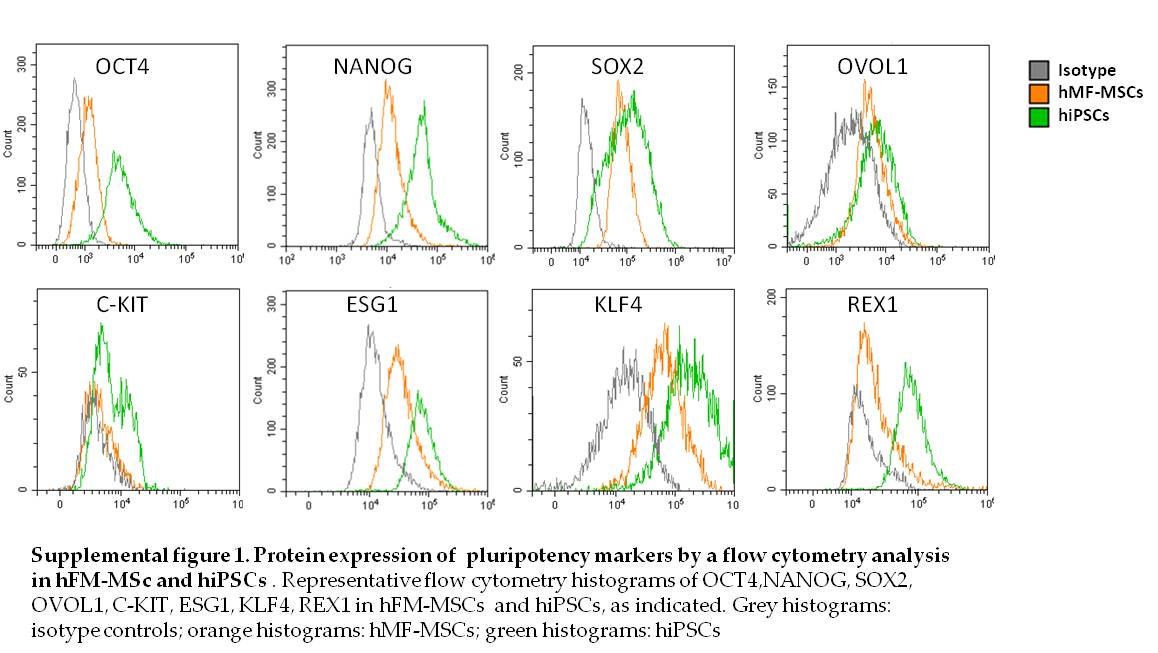

Supplement: Supplementary file 1 [file ijms-21-06589-s001.zip › Revised figure jpeg/Supplemental figure 1.jpg]

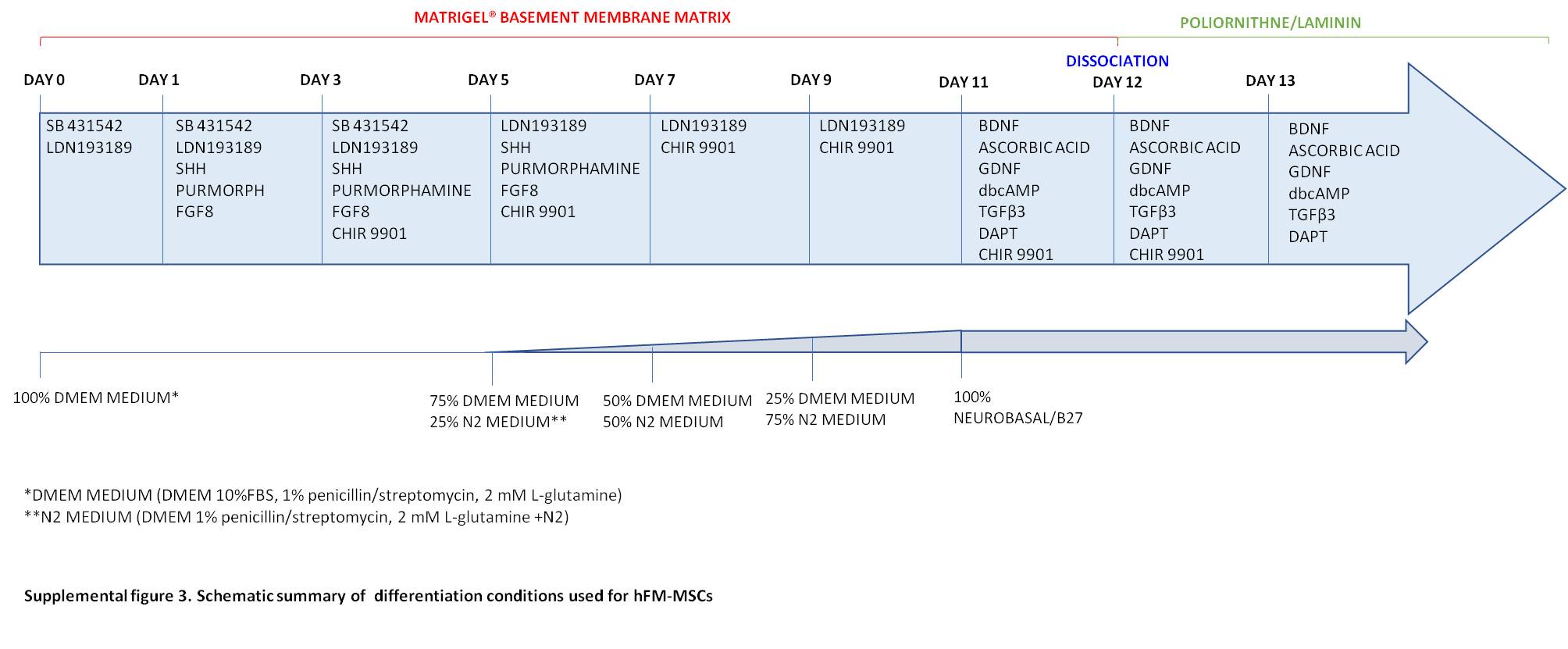

Supplement: Supplementary file 1 [file ijms-21-06589-s001.zip › Revised figure jpeg/Supplemental figure 3.jpg]

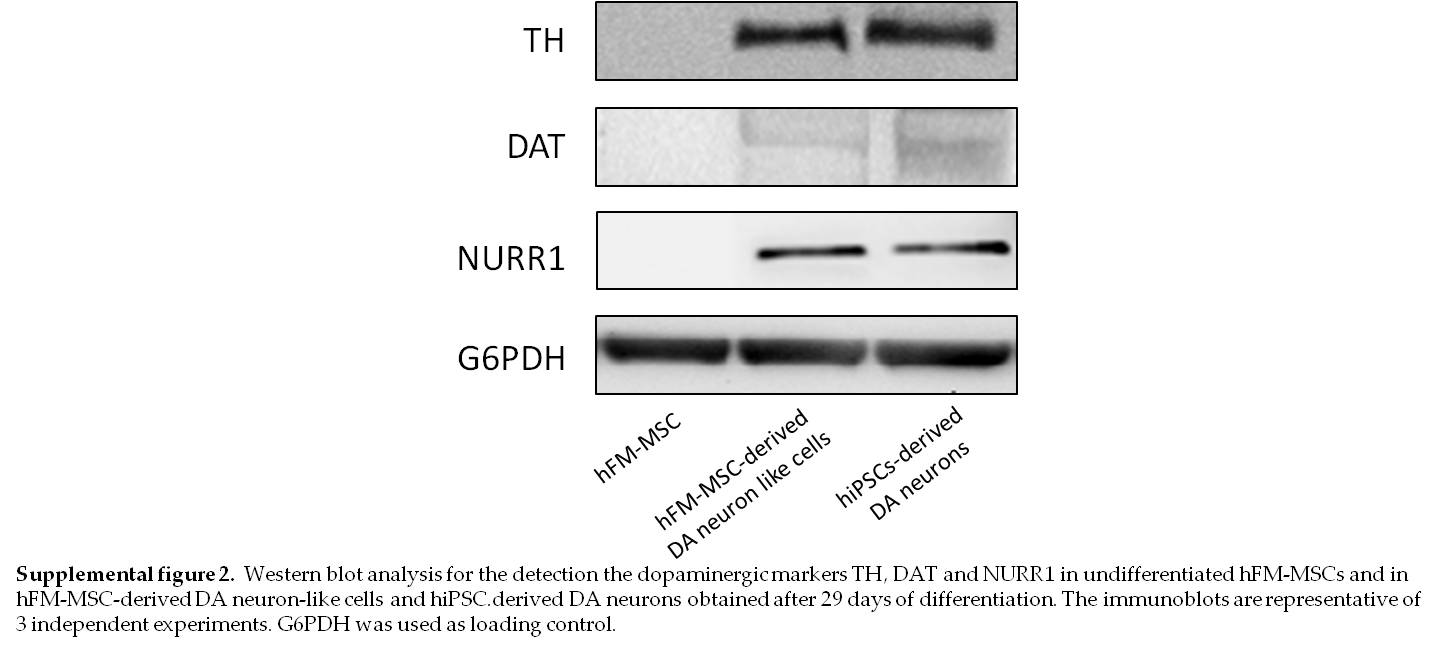

Supplement: Supplementary file 1 [file ijms-21-06589-s001.zip › Revised figure jpeg/Supplemental figure 2.jpg]
